# Biomechanical Performance and Handling of Mineral–Organic Adhesive Bone Cements Based on Magnesium Under Clinical Test Conditions

**DOI:** 10.3390/jcm14093081

**Published:** 2025-04-29

**Authors:** Stefanie Hoelscher-Doht, Alexandra Fabian, Lasse Bögelein, Eva Kupczyk, Rainer H. Meffert, Uwe Gbureck, Tobias Renner

**Affiliations:** 1 Department for Trauma, Hand, Plastic and Reconstructive Surgery, University Hospital Würzburg, Oberdürrbacher Street 6, 97080 Würzburg, Germany; hoelscher_s@ukw.de (S.H.-D.); boegelein_l@ukw.de (L.B.); kupczyk_e@ukw.de (E.K.);; 2 Department for Functional Materials in Medicine and Dentistry, University Hospital Würzburg, Pleicherwall 2, 97070 Würzburg, Germany; gbureck_u@ukw.de; 3 Department of Oral & Maxillofacial Plastic Surgery, University Hospital Würzburg, Pleicherwall 2, 97070 Würzburg, Germany

**Keywords:** bone adhesive, mineral–organic bone adhesive, tibial plateau fracture, biomechanical testing, drillable bone adhesive, porcine fracture model

## Abstract

**Background/Objectives**: Biomineral adhesive bone adhesives composed of phosphoserine combined with magnesium oxides or phosphates exhibit exceptional adhesive properties. This study evaluates two experimental mineral–organic cementitious adhesives in a clinical test setup, investigating their potential for fracture reduction and simultaneous defect filling. **Methods**: The two experimental adhesives (Groups B and C) and a standard hydroxyapatite cement (Group A, reference) underwent compressive strength testing, shear strength testing, and screw pullout tests as part of a first biomechanical characterization. Furthermore, all materials were tested in a porcine tibial split depression fracture model, where they served both for fracture reduction and for filling the metaphyseal bone defect, supplementary to plate osteosynthesis. Fracture stability was assessed under cyclic loading in a materials testing machine. **Results**: The OPLS (O-phospho-L-serine) containing adhesive (Group B) demonstrated the highest compressive strength as well as the highest shear strength. All three materials showed comparable maximum pullout forces. Both experimental adhesives (Groups B and C) exhibited higher pullout stiffness compared to the standard cement (Group A). In the fracture model, no significant differences in displacement under cyclic loading were observed between groups. **Conclusions**: The biomineral adhesive bone adhesives (Groups B and C) demonstrated biomechanical advantages in axial compression, adhesive (shear) strength, and screw fixation compared to the standard hydroxyapatite cement (Group A). Furthermore, they achieved comparable stabilization of metaphyseal fractures under clinically relevant dynamic loading conditions.

## 1. Introduction

The development of a potent bone adhesive is considered a major challenge in terms of materials science. This may be the reason why no bone adhesive has been able to establish itself as a commercially available product yet. A review paper by Farrar et al., 2012 [1], which is now over 10 years old and is often referred to, provides a comprehensive overview of candidate bone adhesives and highlights the significant difficulties involved in their development. These challenges—combining biocompatibility, mechanical strength, adhesion to wet surfaces, and suitable curing times—persist to this day. Additionally, resorbability is a critical requirement to avoid interference with bone healing [1]. Considering that bonding remains difficult even in technical applications, the complexity of developing bone adhesives becomes even more apparent. Further factors such as ease of handling, sterilizability, and cost efficiency also play crucial roles.

While the fundamental challenges have remained unchanged, considerable progress has been made in the development of candidate materials for bone adhesives in recent years. In particular, cementitious materials have attracted attention. Classical bone cements are well-established as synthetic bone substitutes and fulfill core requirements such as biocompatibility and resorbability. However, they are generally not known for adhesive properties and often require demanding curing conditions.

A new class of materials, termed mineral–organic resorbable bone adhesives (MORBA), appears to overcome these limitations. MORBA typically combines organophosphates such as phosphoserine (OPLS) with mineral phases like calcium phosphates. The term MORBA has been used by Picos et al. (2024) [2,3] in the context of ongoing “first-in-man” studies. Although MORBA incorporates components familiar to classical bone cements, they differ fundamentally in their adhesive and setting mechanisms. Calcium phosphates remain among the best-known synthetic bone substitutes [4,5,6]. Moreover, recent research has highlighted the critical role of magnesium in bone regeneration [7,8,9]. Consequently, bioceramics based on magnesium phosphate cements (MPC) have gained attention, offering comparable biocompatibility, higher solubility, and superior mechanical properties compared to calcium phosphate cements (CPC) [10,11,12,13].

Building on this, mineral–organic adhesive bone adhesives combining phosphoserine with magnesium oxides or phosphates have recently been introduced [14]. These materials, which can also be classified as MORBA, demonstrate notably strong adhesive properties. It is hypothesized that the higher oxygen affinity of magnesium compared to calcium contributes to these improved properties [14]. In addition to phosphoserine-based systems, mineral–organic adhesives using phytic acid as an organic component have also been described [15]. It is important to emphasize, however, that existing data are largely based on in vitro experiments under standardized and idealized conditions, which differ considerably from clinical realities.

Despite the emerging optimism, it is unlikely that bone adhesives will initially be used as a standalone treatment for fractures. Instead, they are expected to complement conventional methods, such as by pre-fixating fracture fragments prior to osteosynthesis with plates and screws. Given their biocompatibility and their additional function as bone defect fillers, mineral–organic adhesives may offer clinical benefits during early adoption phases.

In this context, further biomechanical in vitro studies focusing on application-oriented conditions are urgently needed. Particularly relevant aspects include defect filling under fluid exposure, screw anchorage performance, and overall mechanical behavior in adhesive-supported osteosynthesis systems

The primary aim of the present study was to evaluate the mechanical stability and clinical applicability of two promising mineral–organic bone adhesives: (i) a composite containing trimagnesium phosphate hydrate (Mg_3_(PO_4_)_2_·xH_2_O; referred to as TMP·xH_2_O) and O-phospho-L-serine (OPLS), and (ii) a composite based on a farringtonite/stanfieldite-type solid solution (Mg_3_(PO_4_)_2_ and Ca_3_(PO_4_)_2_), magnesium oxide (MgO), phytic acid, and hyaluronic acid. As a reference material, a commercially available hydroxyapatite-forming cement composed of α-tricalcium phosphate (α-Ca_3_(PO_4_)_2_; referred to as α-TCP) and sodium hydrogen phosphate (Na_2_HPO_4_) was used. Specifically, we investigated their ability to withstand screw insertion without compromising stability, their defect-filling capabilities, and their performance in a porcine lateral tibial plateau fracture model. We hypothesized that the two experimental mineral–organic adhesives would achieve biomechanical primary stability comparable to the commercial reference material while additionally offering adhesive properties to support fracture fragment fixation.

## 2. Materials and Methods

### 2.1. Powder Synthesis, Adhesive Production

The cements used are summarized in Table 1. The production of the individual components is described below.

The cement capable of forming HA (group A) was prepared using α-TCP and 2.5% Na_2_HPO_4_. The α-TCP was synthesized by combining 0.716 mol of monetite (Honeywell, Morristown, NJ, USA) with 0.33 mol of calcium carbonate (Merck, Darmstadt, Germany). This mixture was processed in a planetary mill (PM 400, Retsch GmbH, Haan, Germany) with an agate grinding bowl for 1 h before being sintered at 1400 °C. The resulting material was then ground to a particle size of less than 355 µm and subsequently dry-milled for 4 h. To prepare the final cement mixture, a PLR of 3.0 g/mL was used. For application, the powder and liquid components were rapidly mixed on a glass plate using a spatula and immediately transferred into a syringe.

The dry mass of group B cement was formed from the powders stanfieldite/farringtonite (Mg_(3−x)_Ca_(x)_(PO_4_)_2_) and MgO 2933 (Magnesia GmbH, Lüneburg, Germany). The liquids were composed of phytic acid 50 wt-% and hyaluronic acid (Biosynth, Staad, Switzerland). A PLR of 1.714 mg/mL was determined. For the desired synthesis of Mg_2.75_Ca_0.25_(PO_4_)_2_ as cement raw material, 0.167 mol calcium hydrogen phosphate (CaHPO_4_, J.T. Baker, Radnor, PA, USA), 0.083 mol calcium carbonate (CaCO_3_, Merck, Darmstadt, Germany), 1.830 mol magnesium hydrogen phosphate (MgHPO_4_∙3H_2_O, Sigma-Aldrich, Taufkirchen, Germany), and 0.920 magnesium hydroxide (Mg(OH)_2_, VWR International GmbH, Darmstadt, Germany) were sintered at 1100 °C with a holding time of 5 h in each case. The sintered cakes were manually crushed with a mortar and pestle, followed by grinding with four zirconia balls with a diameter of 30 mm each in a planetary ball mill (PM400 Retsch, Haan, Germany) at 200 rpm for 1 h. Afterwards, powders were sieved to a particle size of <125 μm. The adhesive was prepared by mixing the powders Mg_2.75_Ca_0.25_(PO_4_)_2_ and MgO in a powder-to-powder ratio (PPR) of 12.27:1 with a liquid mixture of phytic acid (IP6, 50 wt-%) and hyaluronic acid (17.14 mg/mL) in a liquid-to-liquid ratio (LLR) of 1:1, using a spatula on a glass plate.

For the production of cement group C TMP·xH_2_O powder (Thermo Fisher Scientific GmbH, Dreieich, Germany) was subjected to a heat treatment in the high-temperature oven C 19 (Nabertherm GmbH, Lilienthal, Germany). Initially, the powder was heated to 100 °C over a period of 200 min. After holding this temperature for 30 min, it was further increased to 400 °C within 200 min and maintained at this level for 6 h. The oven was then allowed to cool naturally to room temperature. The adhesive was prepared by manually mixing TMP·xH_2_O and O-phospho-L-serine (OPLS, Sigma-Aldrich GmbH, Steinheim, Germany) in a powder-to-powder ratio (PPR) of 3:8. Water was then added in a powder-to-liquid ratio (PLR) of 2.56 g/mL. Mixing was performed thoroughly and quickly with a spatula on a glass plate until a homogeneous paste was obtained, which was subsequently transferred into a syringe for application.

### 2.2. Mechanical Testing of Raw Materials

**Figure 1:**
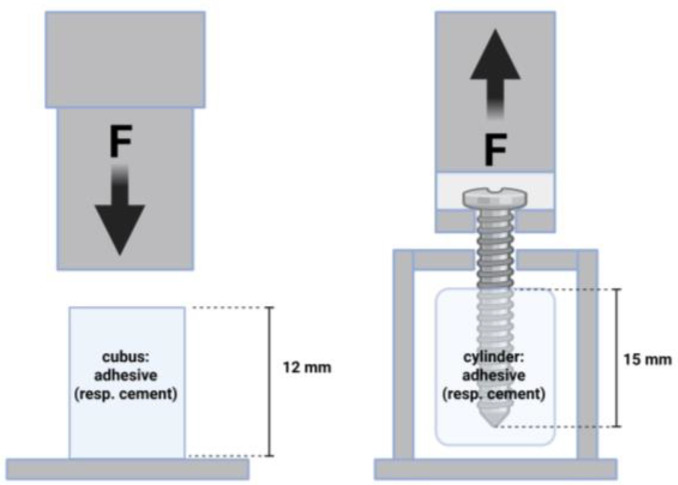
Compression test (**left**) and screw pullout test (**right**) set up.

For material characterization, compressive strength tests were performed. Therefore, cuboid samples measuring 6 × 6 × 12 mm were prepared using silicone molds. After an initial setting period of 15 min at room temperature, the samples were removed from the molds and subsequently incubated in a PBS bath at 37 °C for 24 h. Excess material was carefully removed using abrasive paper, and each sample was remeasured prior to mechanical testing. At least 9 samples per group underwent compressive testing using a universal testing machine (Zwick Z020, ZwickRoell, Ulm, Germany) equipped with a 20 kN load cell. The tests were conducted at a crosshead speed of 1 mm/min (Figure 1).

To analyze the screw pullout force of the different bone adhesives, cylinders (16 mm × 20 mm) were prepared in silicon molds in a special device [16,17]: cement/adhesive cylinders were potted with a central cortical screw (diameter 3.5 mm; length 25 mm; DePuy Synthes, Umkirch, Germany) and cured in a PBS bath for 24 h (*n* = 9 in each case). The screw was embedded in the center at a length of 15 mm. Subsequently, screw pull-out tests were carried out in the Zwicki 2.5 KN materials testing machine (Zwick Roell, Ulm, Germany) with a constant force application. An axial tensile test was performed at a speed of 1 mm/min through the traverse and tubular holder moving upwards to determine the screw pullout (Figure 1). The parameters of interest were the maximum pull-out force and the stiffness.

Shear strength tests were carried out to demonstrate the general differences in adhesion between the two adhesives tested and the hydroxyapatite cement. The technical procedure, including a special device and the technique of sample preparation is described elsewhere [14,18]. Cortical bovine bone was used as the test specimen (*n* = 7 in each case). The tests were carried out 20 min after bonding. The ZwickRoell Z010 universal testing machine (ZwickRoell GmbH & Co. KG, Ulm, Germany) was used with a load cell of 10 kN. From a preload of 1 N, measurements were taken at a test speed of 1 mm/min.

### 2.3. Biomechanical Testing in Fracture Model

#### 2.3.1. Fracture Simulation

A lateral split depression fracture of the tibial head was created in 27 tibias of six-month-old domestic pigs. The lower legs were collected from a local butchery (Metzgerei Hollerbach GmbH, Rimpar, Würzburg, Germany) and were fresh-frozen and wrapped with bandages soaked in physiological saline solution. Prior to testing, the legs were thawed at room temperature (20 °C) for 24 h. In preparation, the tibiae were dissected to separate them from soft tissue. They were then shortened at the distal end to a total length of 20 cm and fixed in a metal device with a valgus angle of 5°, as described in previous studies of our research group, according to the literature [19,20,21], but using Technovit^®^ 5071 as an embedding agent. To simulate a split depression lateral tibial head fracture, analogous to a former project with the evaluation of the fracture model (currently unpublished data), eight 2 mm holes with a depth of 5 mm were drilled in a 12 mm diameter circle at the anterolateral tibial plateau. Two incomplete osteotomies with an angle of 135° to each other, made with a giggly saw and a custom-made template to secure standardization, defined the fracture line.

**Figure 2:**
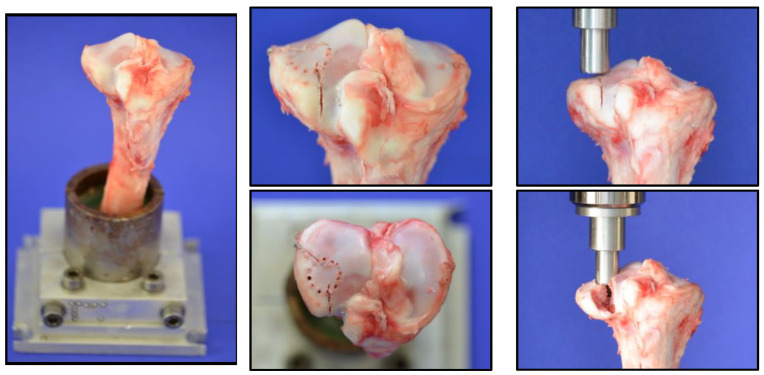
The sequence of induction of a split depressed lateral tibial plateau fracture.

After preparation, the bone was clamped in the Zwick Roell Z020 materials testing machine (ZwickRoell GmbH, Ulm, Germany). After precise alignment of a load stamp over the marked area, the machine simulated the intended fracture with load application at 25 mm/min. The depression depth was limited to 10 mm, while the maximum force applied was recorded using the testXpert III software (V1.3). The process is illustrated in Figure 2.

#### 2.3.2. Fracture Reduction

**Figure 3:**
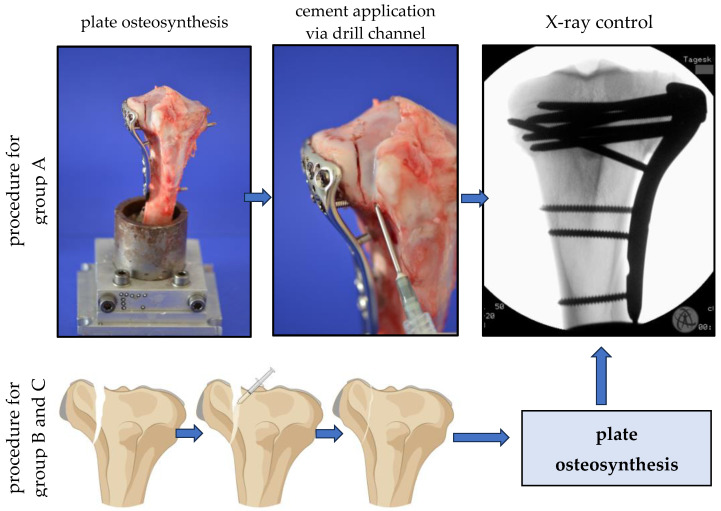
Plate osteosynthesis of a split depressed fracture on the lateral tibial plateau and injection of hydroxyapatite-forming cement and X-ray control (procedure for group A). Gluing of the split depressed fracture on the lateral tibial plateau, plate osteosynthesis, and X-ray control (procedure for groups B and C).

Nine specimens per group were selected randomly into 3 groups, and after the reduction of the simulated split depression fractures, the fractures of the control group (group A) were like the current standard procedure stabilized with a plate osteosynthesis following an application of the hydroxyl apatite forming bone cement (alpha TCP) (see Figure 3). In groups B and C, the fractures were reduced anatomically with the bone adhesive simultaneously filling up the remaining metaphyseal bone defect after lifting of the depressed articular fracture fragment to the articular surface. After first setting the bone glue within 15 min, the same plate osteosynthesis as in group A was performed. As a plate, a typical, often used plate type was chosen (VA-LCP Proximal Tibial Plate 3.5, DePuy Synthes, Johnson&Johnson Medical GmbH, Norderstedt, Germany). All osteosynthesis with the filling of the metaphyseal bone defect was controlled by X-rays before the biomechanical testing. All specimens underwent a hardening in an incubator at 37 °C for 24 h.

#### 2.3.3. Biomechanical Test Set-Up in the Fracture Model

Before starting the biomechanical loading, the bone, the fixation device, and the load indentor were equipped with markers by the optical system (ARAMIS 3D Professional, Carl Zeiss GOM Metrology GmbH, Braunschweig, Germany). The lateral tibial plateau was then loaded cyclically at a constant speed force to simulate repetitive loading by knee joint motion like it is typically for the first timeframe of rehabilitation after surgery. To determine the exact number of cycles and the loading level, besides theoretical considerations, including described test protocols in literature and clinically allowed loading, a pre-testing series was performed with increasing loading levels and cyclic loading up to 10,000 cycles. Finally, the number of 3000 cycles was determined as an interesting range to analyze the motions of the lateral plateau area under loading from 20 N up to 300 N. Ten setting cycles with a load of 20 N to 125 N were carried out before the actual measuring cycles. Loads were applied with a constant speed of 25 mm/min.

During optical data acquisition, every 20th cycle was recorded once the voltage exceeded 2.9 V, resulting in a total of 150 images captured during cyclic loading at 300 N. To analyze movement, self-adhesive markers were applied following a predefined pattern. Prior to marker placement, the bone was evenly coated with white paint to enhance strain measurement, followed by the application of fine black speckles to create a facet component for the optical system. The distance for evaluating the displacement was determined on 3 corresponding pairs of markers, medial and lateral to the fracture gap. For three-dimensional motion analysis, at least three markers per element were used, with four markers applied in certain cases (e.g., at the fracture gap) to improve detection accuracy. The analyzed elements included the prosthetic sled, medial plateau, fracture gaps, and fixation plate. Additional reference markers were placed on the test setup to establish a coordinate system.

**Figure 4:**
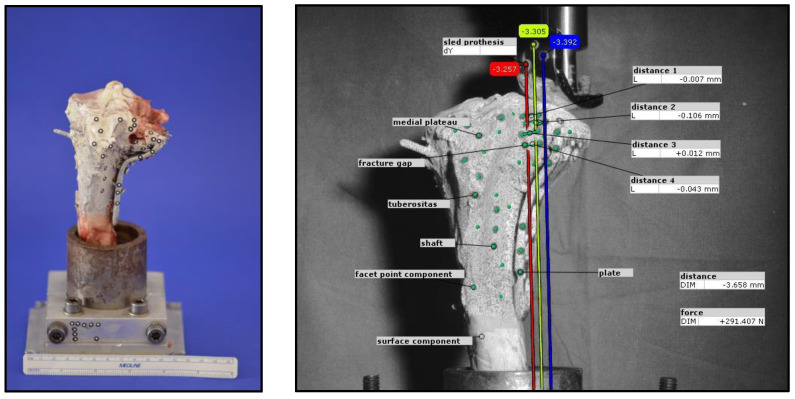
Exemplary test setup for analysis using an optical system.

The camera was positioned laterally to the bone to optimize visualization of the fracture gap. Displacement analysis focused on the y-axis movement of the prosthetic sled within the lateral fracture gap, which was compared to measurements from the materials testing machine. The total displacement at the end of testing was determined as the mean of three y-axis vectors. Furthermore, vertical displacements at four predefined locations within the fracture gap were evaluated. Representative examples illustrating the methodology are shown in Figure 4.

### 2.4. Statistical Analysis

Statistical analysis was conducted following the recommendations of the Institute for Clinical Epidemiology and Biometry (University of Würzburg, Germany) using SPSS v.23 (IBM, New York, NY, USA). The sample size per test and time point was set to *n* = 9. Descriptive statistics were applied to determine mean values and standard deviations. Data distribution was assessed for normality using the Shapiro–Wilk test and the Kolmogorov–Smirnov test, completed with Q–Q diagrams.

If the normal distribution was confirmed, group differences were analyzed using ANOVA or Welch-ANOVA, depending on Levene’s test for homogeneity of variances, followed by Tukey and Games–Howell post hoc tests. In cases where normality was not met, the Kruskal–Wallis test was applied for further analysis. The significance level for all statistical tests was set at 0.05.

## 3. Results

**Figure 5:**
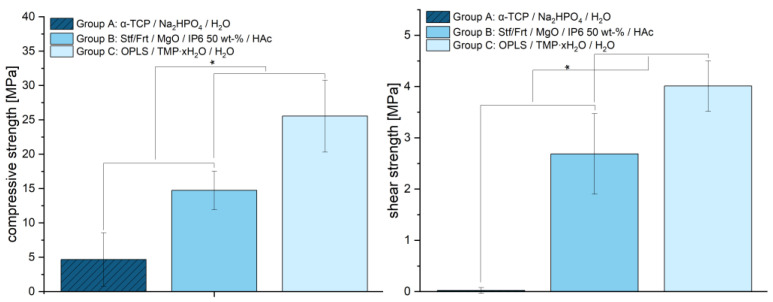
Compressive strength (**left**) and shear strength (**right**) of: Group A (dark blue): α-TCP/Na_2_HPO_4_/H_2_O (reference), Group B (medium blue): stanfieldit/farringtonit (Mg(_3−x_)Ca(_x_)(PO_4_)_2_)/MgO (2933)/IP6 50 wt-%/HAc, Group C (light blue): OPLS/Mg_3_(PO_4_)_2_∙xH_2_O (400 °C heat treated)/H_2_O. Measurement took place after 24 h hardening in PBS solution at 37 °C (*n* = 9, respectively). ANOVA revealed significant differences between groups (*p* < 0.001). Post hoc analysis (Tukey HSD/LSD) confirmed significant differences between all groups (*p* < 0.001). Significant differences are marked with an asterisk (*).

The mechanical properties of the tested materials revealed significant differences in compressive strength, which are shown in Figure 5. The average strength of the samples from group A was 4.66 ± 3.89 MPa, while group B showed a significantly higher strength of 14.73 ± 2.80 MPa (each *p* < 0.001). The highest compressive strength was achieved with group C (25.54 ± 5.23 MPa). Statistical analysis confirmed significant differences between all groups. This indicates that group C has increased mechanical stability due to optimized material composition and stronger crystal structures. With a mean value of 0.02 MPa (±0.06), group A exhibited significantly lower shear strengths than group B (2.69 ± 0.78 MPa) and group C (4.01 ± 0.49 MPa). The differences between the groups indicate that the adhesives in groups B and C provide significantly more adhesion than the hydroxyapatite-forming cement (*p* < 0.001), which exhibits almost no adhesion.

**Figure 6:**
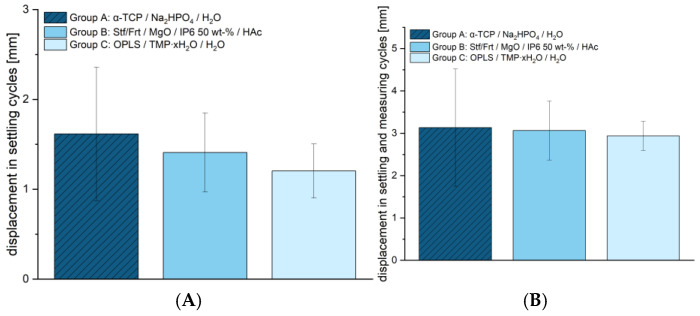
Displacement during settling cycles (**A**) and during settling and measuring cycles (**B**) (*n* = 9, respectively). ANOVA revealed no significant differences between groups in Diagram (**A**) (*p* = 0.27). In Diagram (**B**), ANOVA showed no significant differences between groups A and B (*p* = 0.82), and the Kruskal-Wallis test confirmed the absence of significant differences (*p* = 0.93).

**Figure A1:**
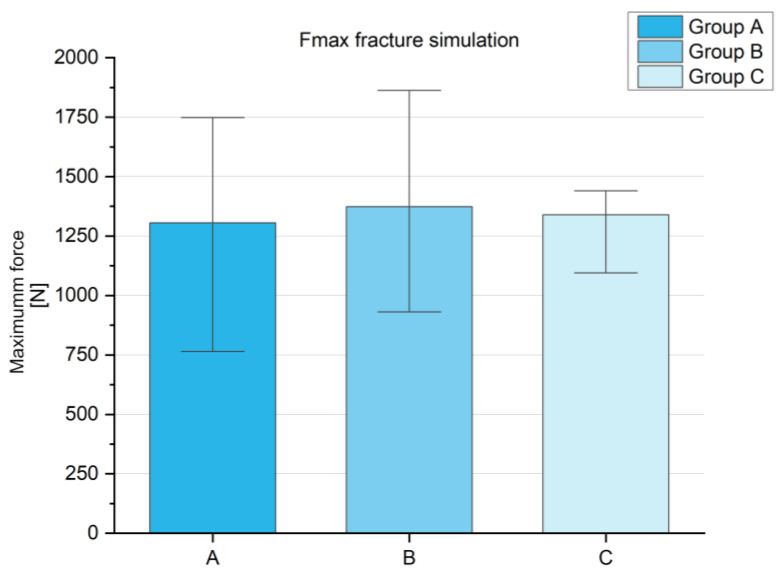
Maximum force in the context of a fracture simulation of a split compression fracture previously treated with an adhesive/cement-supported osteosynthesis (*n* = 9, respectively). ANOVA revealed no significant differences between all groups (*p*-Wert = 0.91).

The maximum force in the fracture simulation (Figure A1, Appendix A) was comparable between the tested groups. Group A showed an average maximum force of 1305.00 ± 309.23 N, group B reached 1373.48 ± 425.43 N, and group C was 1339.73 ± 253.97 N, providing no significant difference between the groups. This is a basic requirement in order to have comparable groups for subsequent studies. The cyclic stress also showed no significant differences between the groups (see Figure 6). Within the setting cycles, deformations of 1.62 ± 0.74 mm were observed for Group A, 1.41 ± 0.44 mm for Group B, and 1.20 ± 0.30 mm for Group C. The total deformation over the setting and measuring cycles also showed similar values with 3.13 ± 1.39 mm for group A, 3.06 ± 0.70 mm for group B, and 2.94 ± 0.34 mm for group C. These results suggest that despite the significant differences in compressive strength, all materials exhibit comparable stability under cyclic loading. The congruent evaluation using an optical system can be found in Table 2.

**Figure 7:**
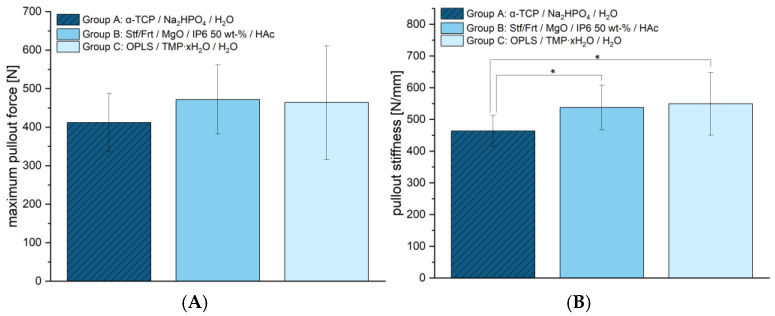
Maximum load (**A**) and stiffness (**B**) of the screws in the context of screw pullout tests (*n* = 9, respectively). Maximum pullout force (**A**) was assessed using ANOVA. No significant differences between the groups were found (*p* = 0.458). Pullout stiffness (**B**) was assessed using ANOVA. Significant differences were found between groups A and B (*p* = 0.048, according to the LSD test) and between groups A and C (*p* = 0.024, according to the LSD test), both marked with asterisks (*). No significant differences were observed between groups B and C.

The maximum force at screw pull-out (see Figure 7A) was determined to show no significant differences between groups. Group A reached 411.78 ± 75.73 N, group B 471.67 ± 89.89 N, and group C 464.00 ± 147.45 N. This indicates that the screw anchors of the new cementitious adhesives have a similar strength to the reference. However, differences were found in terms of stiffness (see Figure 7B). Group A showed the lowest stiffness with 463.71 ± 48.73 N/mm, while Group B with 537.74 ± 70.22 N/mm, and Group C with 549.50 ± 98.80 N/mm achieved significantly higher values. This suggests that the adhesives presented here offer greater resistance to deformation, which could potentially allow for more stable fixation.

In summary, the materials tested demonstrated significant differences in compressive and shear strength, with group C having the highest strength and group A (reference) the lowest in both cases. Despite these differences, the materials were comparable in the fracture simulation and under cyclic loading, suggesting that other mechanical factors play a role. The screw pull-out tests showed no differences in maximum force but a higher stiffness for group C compared to the reference.

## 4. Discussion

In general, there is still little literature addressing adhesively supported osteosynthesis. Smeets et al. (2010, 2013) [22,23] used modified PMMA and an amphiphilic adhesive agent in both in vitro and in vivo studies. However, the basic idea of hybrid procedures using bone adhesives and conventional osteosynthesis material differs fundamentally in the technical procedure and in the intention of the study presented here. Smeets et al. (2010, 2013) [22,23] glued osteosynthetic plates without subsequently screwing them in place. This may be indicated for fragile bone flaps, such as midface fractures, where a screw can hardly find a hold. In the present study, however, the adhesive was inserted into the fracture gap. On the one hand, this can serve to prefix displaced fragments before osteosynthesis and support osteosynthesis in the subsequent procedure. On the other hand, it can be used to fill osseous defects within the fracture gap, as bone adhesives are also bone replacement materials in this respect. Prominent bone cements such as PMMA are unsuitable for such a procedure. On the one hand, focal necrosis [24,25] and mild to severe foreign body reactions [24,25,26] have been described when using PMMA, albeit in comparatively older literature, which could be considered critical when used in the fracture gap. On the other hand, PMMA is generally not absorbable [27,28]. Although PMMA is described to induce membranes that could potentially facilitate bone healing through its biological properties [29,30,31], PMMA itself may act as a physical barrier [27], which would have serious consequences if used in a fracture gap. Calcium phosphate bone cements, on the other hand, have shown promising results in the treatment of fractures. A meta-analysis found that the use of calcium phosphate is associated with less pain at the fracture site and a lower prevalence of fracture remission compared to conventional treatments [32]. These materials act as synthetic osteoconductive scaffolds and promote direct osteoid formation on their surface [33]. In vivo studies in animal patients support the hypothesis that calcium phosphate cement, in combination with plate osteosynthesis, can promote bone healing in problem patients [34].

At this point, it should be clearly pointed out that the new bone adhesives presented here do have cement-like properties and workability. However, even if they include some traditional cement phases, they are not conventional cements from a materials science perspective. They are furthermore based on magnesium phosphate or magnesium oxide phases, which is unconventional. It should also be emphasized that the above-mentioned cements, whether PMMA or calcium phosphate cements, presumably have no significant adhesive properties and have not been developed for this purpose. To the authors’ knowledge, biomechanical tests on declared bone glues in the context of adhesively supported augmenting osteosynthesis have been carried out for the first time, which complicates direct comparisons with other studies.

At 25.54 ± 5.23 MPa, the tested bone adhesive consisting of phosphoserine and magnesium phosphate hydrate (group C) showed a significantly higher compressive strength than the calcium phosphate cement used (reference, group A). This determined compressive strength was consistent with results from the literature on this adhesive, with values between 19.25 ± 0.88 MPa and 34.82 ± 2.03 MPa, depending on the time of measurement (up to 7 days) [14]. No published data are yet available for group B. With regard to compressive strength, however, it was also shown that this did not correlate with high adhesive strength [14]. Rather, the decisive factors for a successful bond are the surface quality and wettability of the parts to be joined [35]. However, high compressive strength may become important if the bone is augmented with the adhesive in addition to the bonding itself. In spite of this, the investigations on shear strength (see Figure 5) indicated clear adhesive properties compared to the conventional hydroxyapatite-forming cement, which showed almost no adhesion. Although previous studies demonstrated even higher shear strengths [14], the adhesive of group C was superior to the other materials in the work presented here. Although the same methods for pure material tests were used, there may be many reasons for differences between studies, which, in the authors’ opinion, is quite typical, as the determination of adhesion is subject to a large number of uncertainty factors [1]. In this case, inter-individual differences in bone quality, operator-dependent variability in a manual mixing process with a glass plate and spatula, or the moisture content of the bone could have been decisive factors. At 2.94 ± 0.34 mm, the total displacement of group C (phosphoserine and magnesium phosphate hydrate) did not differ significantly from the other groups tested. Evaluations of the optical system (see Table 2) support the displacement values determined. In a similar fracture model on the tibial plateau, Heilig et al., 2021 [17] determined total displacements between approx. 1.4–2 mm [17]. However, their study utilized Synbone^®^ 1110 (Synbone, Malans, Switzerland), a polyurethane-based synthetic model designed to mimic human bone, whereas real bone was used in the present study. While Synbone^®^ provides a standardized and reproducible test environment—which is why it is often used in in vitro studies [36]—it lacks the complex biological variability of real bone, all of which can influence mechanical behavior. The differences in displacement may also be due to the interaction between bone cement/adhesive and the surrounding material. Synthetic bones generally lack the appropriate moisture content. Yet moisture on the bone surface is often a key problem for potential bone adhesives [1]. An optimal bond is primarily achieved by selecting an adhesive that best matches the substrate material and the expected load conditions [37]. Since Synbone^®^ is made of polyurethane rather than real bone, adhesives that adhere well to Synbone^®^ may not necessarily exhibit the same bonding properties on actual bone, and vice versa. Finally, it is important to emphasize that, in this study, a significantly more complex impression depression fracture was induced rather than a simple compression fracture and a different osteosynthesis technique was employed. So, even if a similar test setup was used, the comparability in such a complex test setup is limited. Either way, displacement is of particular interest because residual incongruity in intraarticular fractures has been linked to the development of posttraumatic arthrosis [38]. Since articular step-offs up to 3 mm were found to have no adverse effects on clinical outcomes in tibial condyle fractures in the longer-standing study by Honkonen (1994) [39], the displacement observed in this study, which remained within this range across all groups, can still be considered acceptable. It should also be mentioned in this context that bone adhesives, in contrast to conventional cements, can contribute to improving the reduction outcome. In particular, they have the potential to primarily stabilize the fracture and fix the fragments in an anatomically correct position. During the application of the osteosynthesis material and subsequently, micromovements can presumably be reduced. The bone adhesives tested showed a maximum pullout force of approx. 1300–1370 N in the screw pull-out test. These values were, on one hand, comparable to those of the established reference (group A). On the other hand, they were similar to the pullout strengths of experimental drillable magnesium phosphate cements, although they were slightly inferior in comparison. In in vitro bone models, 10 ± 0 N to 1150 ± 60 N were described, depending on the insertion angle, bone type and quality [40]. In light of these results, the authors assume that screws can be effectively anchored in the here examined bone adhesives. Future studies should also investigate the performance of the adhesive bone cements in osteoporotic bone, where reduced bone density and altered remodeling dynamics may affect bonding and degradation behavior. Particular attention should also be given to bone under antiresorptive therapy, as changes in turnover rates could influence the integration and longevity of the adhesive interface.

In summary, it can be said that the two bone adhesives investigated offer not only adhesive properties but also biomechanical advantages in terms of compressive strength and screw pull-out strength in contrast to the established hydroxyapatite-forming cement. On the other hand, they showed acceptable displacement similar to that of established hydroxyapatite cement at postoperatively relevant load.

Apart from biomechanical parameters, parameters such as biocompatibility, osteoconductivity, or osteogenic potential play an important role. The new mineral–organic bone adhesives, which contain organophosphates, have been less studied in this respect than conventional bone cements. However, it can be assumed that they have a high osteogenic potential. Phosphatases of amino acids such as serine or threonine play an important role in the regulation of proliferation, differentiation, maintenance, and function of osteoclasts [41], although the explicit role of organophosphates relevant for bone adhesion, such as phosphoserine, is not yet fully understood. Phosphoserine (the main component in group C) stimulates osteoblast differentiation of MC3T3-E1 cells when added to the cell culture medium and has been shown to significantly enhance bone formation in a rabbit defect model when incorporated into hyaluronic acid [42]. Similarly, CPCs containing phosphoserine promoted increased bone remodeling and formation in rats, particularly at the early stages of bone healing [43]. Additionally, studies have reported that phosphoserine accelerates the resorption of calcium phosphate cements in mini pigs [44]. Kirillova et al. (2018) showed that a bone adhesive containing phosphoserine as its main component, which is explicitly declared for this purpose, involves both bone healing and osteointegration [45]. Hulsart-Billström et al. (2020) [46] also showed that a novel bone adhesive containing phosphoserine is biocompatible and has no harmful effects on the surrounding tissue. In addition to phosphoserine, phytic acid (the main component in group B) is still less prominent in the use of bone adhesives. However, there are indications that phytic acid can be used as a useful setting retardant and, at the same time, evokes comparatively higher cell proliferation and activity [47].

In this context, one limitation of the biomechanical porcine tibia model used in this study is that it does not allow for conclusions regarding the biocompatibility or long-term biological response to the tested bone cements. Moreover, its transferability to clinical fracture treatment in humans is limited, particularly concerning osteoporotic fractures, as young porcine bone tissue was used. Additionally, factors such as natural bone healing and soft tissue interactions cannot be replicated. Nevertheless, the model provides valuable insights into the mechanical properties and initial stability of the materials under standardized conditions. The findings can serve as a foundation for further in vivo investigations.

## 5. Conclusions

Overall and in conclusion, one must say that the investigated bone adhesives exhibit not only adhesive properties but also demonstrate significant biomechanical advantages over conventional hydroxyapatite-based cements. In particular, they show superior compressive strength and screw pull-out resistance while maintaining displacement behavior within clinically acceptable limits. Especially the composition of magnesium phosphate hydrates and phosphoserine (group C) could be a promising choice for fracture fixation due to its superior properties and effectiveness as a bone filler. In general, our findings suggest that bone adhesives can contribute to improved fracture stabilization by providing primary fixation and reducing micromovements during osteosynthesis. While additional research is needed to explore their full biological potential, existing literature indicates that components such as phosphoserine may support bone remodeling and integration. However, the primary significance of this study lies in the biomechanical evaluation, which highlights the potential of bone adhesives as a valuable addition to augmenting osteosynthesis techniques. Further research should focus on long-term in vivo studies to assess biological integration, degradation behavior, and overall impact on bone healing.

## Figures and Tables

**Table 1:** Summary of the composition of the cements or cementitious adhesives used. Where necessary, concentrations and the powder-to-liquid ratio (PLR), the powder-to-powder ratio (PPR), and the liquid-to-liquid ratio (LLR) are also indicated.

	Group A	Group B		Group C	
	**material**	**material**	**PPR**	**material**	**PPR**
**powder 1**	(1): α-TCP	(1): stanfieldite/farringtonite (Mg_(2.75)_Ca_(0.25)_(PO_4_)_2_)	$\frac{12.27}{1}$	(1): OPLS	$\frac{3}{8}$
**powder 2**	(2): -	(2): MgO 2933		(2): TMP∙xH_2_O	
			**LLR**		
**liquid 1**	(1): Na_2_HPO_4_	(1): IP6 50 wt-%	$\frac{1}{1}$	(1): H_2_O	
**liquid 2**	(2): -	(2): hyaluronic acid (17.14 mg/mL)		(2): -	
**PLR**	**3.0 g/mL**	**1.71 g/mL**		**2.56 g/mL**	

**Table 2:** Displacement in the setting and measuring cycles, measured with the optical system.

Group	Mean Value	Normally Distributed	Statistical Analysis
group A	3.0046 ± 1.4409 mm	yes	ANOVA and Kruskal–Wallis: no significant differences (*p* = 0.82; *p* = 0.93)
group B	2.8189 ± 0.8547 mm	yes	
group C	2.7113 ± 0.2443 mm	no	-

## Data Availability

The data can be made available upon request from the corresponding author.

## Abbreviations

The following abbreviations are used in this manuscript:

CPC	calcium phosphate cement
IP6	inositol hexakisphosphate (phytic acid)
LLR	liquid-to-liquid ratio
MPC	magnesium phosphate cement
MORBA	mineral organic resorbable bone adhesive
PBS	phosphate-buffered saline
PLR	powder-to-liquid ratio
PPR	powder-to-powder ratio
TMP	trimagnesium phosphate
